# The Role of Life History Questionnaires in Defining Individualised Goals of Care for Clinical Cognitive Motor Dissociation Patients: A Pilot Study

**DOI:** 10.3390/brainsci15030267

**Published:** 2025-03-01

**Authors:** Yago Rodriguez Mateos, Karin Diserens, Jennyfer Becquet, Etienne Rochat, Ralf J. Jox, Ivo A. Meyer

**Affiliations:** 1 Neurorehabilitation Unit, Department of Clinical Neurosciences, Lausanne University Hospital and University of Lausanne, 1011 Lausanne, Switzerland; karin.diserens@chuv.ch (K.D.); jennyfer.becquet@chuv.ch (J.B.); meyer.ivo@gmail.com (I.A.M.); 2 Institute of Humanities in Medicine, Lausanne University Hospital and University of Lausanne, 1007 Lausanne, Switzerland; etienne.rochat@chuv.ch (E.R.); ralf.jox@chuv.ch (R.J.J.); 3 Palliative and Supportive Care Service, Department of Medicine, Lausanne University Hospital and University of Lausanne, 1011 Lausanne, Switzerland; 4 University Hospital of Psychiatry, University of Bern, 3000 Bern, Switzerland

**Keywords:** disorders of consciousness, personal narratives as topic, life change events, neurological rehabilitation, motivation, patient care team

## Abstract

Background/Objectives: The aim of this pilot study is to qualitatively describe the use of life history questionnaires in an acute neurorehabilitation setting to define individualised goals of care for patients with clinical cognitive motor dissociation and to determine to what extent the information they contain influences the care and management provided by the team. Methods: Using the patient records of our sample, all individualised goals of care were summarised, which were subsequently used to assess whether life history questionnaires had supplied sufficient information to define various individualised goals of care. We then conducted semi-structured interviews with the involved healthcare professionals to gain insights on how these questionnaires were used. Results: Approximately one-third of all individualised goals of care belonging to the “Activities and Participation” ICF category were defined through life history questionnaires. The semi-structured interviews highlighted the impact of these questionnaires in three main aspects: creating a therapeutic alliance with the patient, facilitating multidisciplinary cohesion, and nurturing a sense of empathy amongst the healthcare professionals. Conclusions: Life history questionnaires are an important contribution to individualised goals of care and may improve the relationship with the patient and the therapeutic setting for all healthcare professionals involved.

## 1. Introduction

### 1.1. The Unresponsive Brain-Injured Patient

While the field of neurology and psychology has seen technological advancements, the definition of consciousness remains a widely debated subject. The most widely used definition of consciousness divides the concept into two components: awareness (content of consciousness) and arousal (wakefulness). Disorders of consciousness (DOCs) arise from deficits in these components [1]. Various clinical states exist on a continuum, ranging from coma [2], to Unresponsive Wakefulness State (UWS), a term that replaced Vegetative State [3] to avoid its potentially pejorative connotation [4], to Minimally Conscious State (MCS) and emergence from MCS (eMCS) [5], each with unique characteristics. MCS has been further divided into two classifications: Minimally Conscious State-Minus (MCS-) and Minimally Conscious State-Plus (MCS+). The MCS- category shows sporadic but discernible signs of consciousness, such as visual tracking, while the MCS+ category represents more advanced behaviours, like responding to commands or producing intelligible speech [6].

However, recent discoveries indicate that many patients, seemingly unresponsive, might possess cognitive abilities beyond traditional definitions of DOC [7,8]. A term gaining acceptance for this state is cognitive motor dissociation (CMD) [9], which describes patients whose brain activity, observable through advanced imaging (especially functional MRI [fMRI]) and electrophysiology (EEG) techniques, does not result in motor responses for communication (i.e., classified as UWS or MCS-). Essentially, their brains can generate signals consistent with language comprehension, but a disconnection between cognition and motor behaviour prevents them from showing it on a behavioural level. This distinction is vital for patient management, as CMD patients tend to have considerably better recovery paths [10].

The recommended clinical assessment scale, the Coma Recovery Scale-Revised (CRS-R) [11], may misdiagnose at least 15% [10] and more likely up to 30% of these patients [12], as it was not designed to detect CMD. Consequently, many patients may possess greater cognitive capabilities than indicated by the CRS-R diagnosis. This has been demonstrated, for instance, with eye-tracking technology, which can detect subtle ocular responses in some patients classified as UWS, suggesting residual higher-order cognitive processing [13]. To systematically address this, the Motor Behaviour Tool-revised (MBT-r) [14,15] was developed. It assesses patients for subtle signs of intentionality—potentially missed by the CRS-R—while ensuring the absence of brainstem disinhibition, i.e., clinical signs suggestive of extensive bilateral damage linked to poor outcomes. Such patients, categorised as non-communicative based on the CRS-R, exhibit recovery paths similar to those of fMRI/EEG-diagnosed CMD. This condition is termed clinical CMD (cCMD). While there is a certain overlap between fMRI/EEG-diagnosed CMD and cCMD, genuine covert awareness patients (representing the purest form of CMD) would not display any detectable signs during bedside clinical examinations. In contrast, cCMD encompasses patients who might show subtle awareness signs that language-based fMRI/EEG paradigms miss due to attentional or language impairments [16].

The clinical assessment of an unresponsive patient in the acute phase demands a comprehensive examination approach, including the identification of both clinical and paraclinical factors influencing conscious behaviour, i.e., pitfalls (such as aphasia, polyneuropathy, akinetic mutism, etc.) that may affect motor interaction [17]. As a rule of thumb, if pitfalls are detected, a diagnosis of cCMD is more likely, unless signs of brainstem disinhibition on MBT-r are detected.

### 1.2. The Importance of a Motivational Framework

It is known that the motivational framework is rooted in the mesolimbic and nigrostriatal pathways. These dopaminergic pathways, connected to motivation and habits, project to the motor thalamus, are termed a superintegrator [18]. This region enhances motor execution and interacts with motor and premotor areas. Damage to thalamo-cortical fibres, detected in CMD patients, offers a potential explanation for limited motor responses despite largely intact cognition [19]. The diagnostic utility of the MBT-r is underscored by the fact that the improvement of the motivational context (e.g., mother tongue, patient’s own name) is specifically one of the items to improve detection of near-covert consciousness [15].

On a therapeutic level, the neurosensory approach implemented in the acute neurorehabilitation setting aims at soliciting all sensory modalities in order to re-establish the thalamo-cortical excitatory coupling and allow the patient to recover functionally. Stimuli with a salient emotional component are preferentially integrated into the cognitive system and are therefore more likely to capture the patient’s attention [18,20,21]. For instance, using the patient’s given name has been shown to elicit a measurable increase in the P300 (P3) response [22,23]. Another example to illustrate this motivational context is swallowing rehabilitation. The early, controlled use of pleasurable food and taste stimuli can improve swallowing and likely expedite decannulation ([24], p. 92) and [25]. The motivational context is a significant factor in executive function. Personalised sensory stimuli, such as outdoor neurosensory stimulation and exposure to natural environments, can enhance goal-oriented behaviours and reduce stress levels in patients with severe brain injuries [26,27]. Motivation and family support may be even more important to the outcome than the patient’s level of functioning [28]. Thus, there is a specific motivational framework for each patient and for each functional recovery goal/objective.

Nevertheless, the motivational framework may be difficult to define in the context of a non-communicative patient. The patient’s family and friends should be involved and become an integral part of the management by providing relevant information to define the motivational framework. In order to assess and evaluate the patient’s individual goals of care throughout the hospital stay, a common tool must be used to define individual goals and determine whether or not these have been achieved [29].

### 1.3. Individualised Goals of Care and Life History Questionnaires

The International Classification of Functioning, Disability and Health (ICF) is a framework and classification system that allows any functional impairment or disability to be classified and assessed in three main categories to distinguish the extent of impairment. The three categories are “(A) Body Structures”, “(B) Activities and Participation” and “(C) Environmental Factors” [30]. In this context, the objectives are called individualised goals of care.

As plausible as it may seem that incorporating individualised goals of care could enhance patient and caregiver engagement while ensuring that rehabilitation goals are met, evidence supporting the overall impact of this goal-setting on rehabilitation outcomes for adults with various disabilities, including brain injuries, remains limited. The strongest available evidence suggests that any potential benefits are primarily in psychosocial outcomes, such as emotional well-being and health-related quality of life, rather than in physical recovery [31,32].

In this context, we developed a life history questionnaire as a tool to complement ICF-based individualised goals of care by systematically integrating patient-specific information in a reproductible way. Structured using a modified S.M.A.R.T. objective system—comprising Specific, Measurable, Achievable (replacing Assignable), Relevant (replacing Realistic), and Timed categories [33]—our questionnaire systematically documents a patient’s social and professional background, hobbies, habits, and interests. By incorporating insights from family members or close friends, it aims to establish a motivational framework that accounts for all available psychosocial dimensions, potentially bridging the gap between standardised rehabilitation approaches and truly personalised care. To our knowledge, no studies on disorders of consciousness have explored the integration of family-reported life history—via questionnaires or other methods—into the neurorehabilitation process.

The objective of this study is to qualitatively assess the utility of life history questionnaires in defining ICF-based individualised goals of care for patients with cCMD, although disorders of consciousness were not formally excluded. Additionally, the study aims to determine the extent to which the information contained in these questionnaires influences the care and management provided by the acute neurorehabilitation team, as assessed through semi-structured interviews.

## 2. Materials and Methods

### 2.1. Study Design

This is a retrospective study designed to assess the implementation of life history questionnaires in defining individualised goals of care for cCMD patients in an acute neurorehabilitation setting and to determine to what extent the information they contain influences the care and management provided by the interdisciplinary team.

### 2.2. Study Population

Inclusion criteria comprised all consecutive patients admitted to the Acute Neurorehabilitation Service at Lausanne University Hospital (CHUV) between July 2020 and January 2021 with any brain injury (e.g., traumatic brain injury, subarachnoid haemorrhage). All patients were initially in an unresponsive state during the first evaluation in the intensive care unit.

Exclusion criteria included patients who were not admitted to the acute neurorehabilitation unit, those without available family members or caregivers, or cases where family members or caregivers refused to complete the life history questionnaires.

### 2.3. Life History Questionnaires

As part of the admission procedure, occupational therapists were tasked with conducting in-depth interviews with the patient’s relatives to complete the life history questionnaires.

### 2.4. Goal Setting and Evaluation

Individualised goals of care were set upon admission and re-evaluated weekly throughout the hospital stay.

These goals were categorised according to the International Classification of Functioning, Disability and Health (ICF) and grouped into three main categories: (A) Body Structures, (B) Activities and Participation, and (C) Environmental Factors.

### 2.5. Assessment of the Life History Questionnaires’ Utility

Using patient records, all individualised goals of care were summarised in a spreadsheet, which was subsequently used to assess whether the questionnaires had supplied sufficient information to outline various individualised goals of care.

To document the progress in functional recovery, we utilised the following rating scales: the Coma Recovery Scale-Revised (CRS-R) [11] to evaluate consciousness levels and the Motor Behaviour Tool-revised (MBT-r) [14,15] to identify subtle signs of intact consciousness and the absence of brainstem release signs, which are necessary for the diagnosis of cCMD. Additionally, we used the Barthel Index [34] to assess functional independence, the Disability Rating Scale (DRS) [35] for global disability assessment, the Functional Ambulation Categories (FAC) [36] for gait evaluation, the Glasgow Outcome Scale (GOS) [37] for overall neurological outcomes, and the Rancho Los Amigos Scale (RLAS) [38] to assess cognitive and behavioural recovery. These scales were selected based on their established validity and relevance in evaluating recovery trajectories in patients with disorders of consciousness.

### 2.6. Semi-Structured Interviews

Semi-structured interviews were conducted with members of the multidisciplinary team, including two physiotherapists, three occupational therapists, a physician, three nurses, a neuropsychologist, and a spiritual advisor. Specific questions asked are listed in Table S2.

The goal of the interviews was to gain insights into the practical utilisation of the life history questionnaires in patient management and their impact on defining goals of care.

### 2.7. Data Analysis

Data from the life history questionnaires and individualised goals of care were compiled and analysed.

The analysis focused on categorising goals in relation to the ICF framework and identifying the contribution of life history questionnaires, particularly within the “Activities and Participation” category.

### 2.8. Ethical Considerations

The study was approved by the Ethics Committee Vaud (CER-VD), and written informed consent was obtained from patients or their legal surrogates to participate in the study.

AI-assisted technology (OpenAI ChatGPT-4o) was used for language refinement and readability improvement at the end of the writing process. It was not used to generate scientific insights, analyse data, interpret findings, or draw conclusions.

## 3. Results

### 3.1. Individualised Goals of Care

A sample of ten patients, including seven males and three females, was analysed. All patients were diagnosed as suffering from a clinical CMD. Patients with true disorders of consciousness were not formally excluded from the study; however, no such patients were present during the data collection phase. In fact, patients with true disorders of consciousness had only constituted a small minority of those hospitalised in our ward [12]. All patients benefited from a neurosensorial approach, involving the collaborative efforts of all caregivers and therapists of the team.

Patient details are listed in Table 1. Life history questionnaires for each of them were collected from their next of kin by occupational therapists. The average duration of hospitalisation in the acute neurorehabilitation unit was 23 days. Cause of brain impairment varied amongst patients, with four due to traumatic brain injuries, two to subarachnoid haemorrhage, two to encephalopathy, and two to a stroke. A total of 251 International Classification of Functioning (ICF) individualised goals of care were identified and scored.

**Figure 1:**
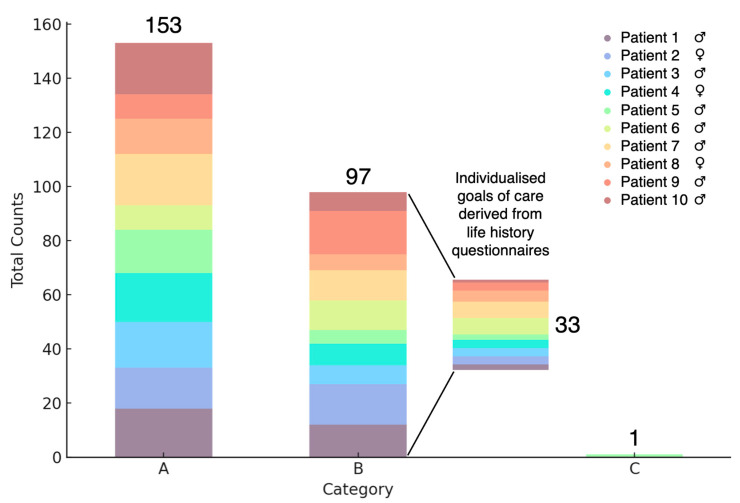
Distribution of individualised goals of care Across ICF Categories (Total = 251). The height of each segment of the stacked bar corresponds to the number of individualised goals of care, and each colour corresponds to a specific patient. For a total of 10 patients, 251 individualised goals of care were defined. Of these, 13% were derived from life history questionnaires, with 34% specifically contributing to category B. International Classification of Functioning (ICF) main categories: A = Body Structures, B = Activities and Participation, C = Environmental Factors.

From a total of 251 individualised goals of care assessed for the ten patients in our study, information from life history questionnaires contributed to the definition of 33 objectives (Figure 1), representing 13% of the total. All these objectives fell into the category “(B) Activities and Participation”. When considering only the objectives within this category, the questionnaires played a role in defining 34% of them. On the other hand, the questionnaires were used by all healthcare professionals involved in the study as an effective approach to support the individualised goals of care, even when they did not directly contribute to defining them.

**Figure 2:**
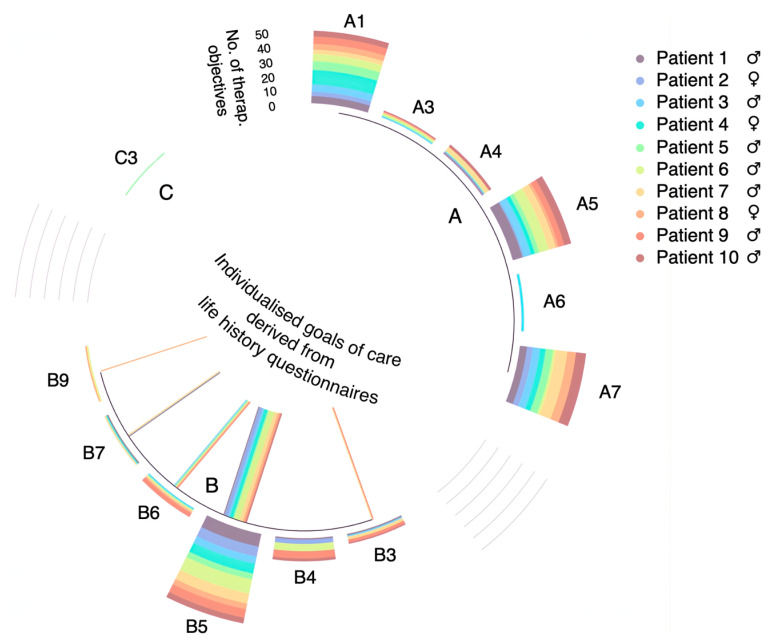
Individualised goals of care by ICF category and life history questionnaire contributions. Individualised goals of care are categorised by International Classification of Functioning (ICF), indicated on the perimeter of the circle; life history-derived objectives are represented by lines, the height of each segment of the stacked bar and the width of each line correspond to the number of individualised goals of care, and each colour corresponds to a specific patient. Categories to which life history questionnaires have contributed: B3: Communication, B5: Self Care, B6: Domestic Life, B7: Interpersonal interactions and relationships, B9: Community, social and civic life.

We have analysed the distribution of the 33 individualised goals of care defined through life history questionnaires according to the ICF subcategories (Figure 2). Sixty-six percent of these individualised goals of care concern Personal Maintenance (B5). Examples of their application are the use of manual or electric razors, make-up or the application of perfume (refer to Supplementary Table S3 for an illustrative example). Fifteen percent of the individualised goals of care defined through the questionnaires concern activities related to “Domestic Life” (B6). These individualised goals of care refer to the preparation of drinks that the patient enjoys (coffee, tea, with or without sugar) and the preparation of meals for patients who used to make them themselves, either for their own consumption or for their relatives.

### 3.2. Semi-Structured Interviews Analysis

We conducted semi-structured interviews with all 11 caregivers and therapists managing the 10 patients.

Life history questionnaires were not utilised by physiotherapists or neuropsychologists to define individualised goals of care, as they relied on their own standardised test protocols. In contrast, occupational therapists systematically incorporated these questionnaires to identify items from the ICF category “(B) Activities and Participation”. As previously mentioned, 34% of the individualised goals of care were derived from information provided in the questionnaires.

All eleven members of the multidisciplinary team reported using life history questionnaires to establish a bond of trust with patients and their families, as well as to define the motivational framework for care. Seven members highlighted that these questionnaires improved multidisciplinary cohesion, while eight noted that they contributed to a more rewarding working environment. Additionally, nine members identified life history questionnaires as a valuable tool for defining individualised goals of care.

## 4. Discussion

In this section, we will discuss the originality of the life history questionnaires as well as the results obtained during our analysis, in particular the discrepancies between their supposed use and their actual application in the clinical context of patient care.

### 4.1. Originality of the Life History Questionnaires

Life history questionnaires provide a wider description of the patient’s life prior to the injury, incorporating personality traits, spiritual beliefs and salient events. We did not find evidence of any other tool used in a multidisciplinary neurorehabilitation care aiming to provide such extensive and individualised information about the patient. According to this perspective, they are an innovative tool, which can be used by various parties involved in acute neurorehabilitation care. In patients with prolonged disorders of consciousness, detailed questionnaires have been developed to assess multidimensional aspects of their condition, including recovery trajectories, ethical considerations, and the impact of intensive neurorehabilitation [39,40]. However, these tools focus on the patient’s current state rather than their pre-injury life history and thus do not serve the purpose of tailoring the neurorehabilitation process. Although distinct from our project, the SAFIR-e initiative focuses on integrating and supporting the families of patients with acute brain injury, highlighting the importance of involving relatives in the care process [41].

### 4.2. Challenges in Defining and Documenting Individualised Care Goals

The findings related to the initial aim—using life history questionnaires as a tool to define individualised goals of care—were only partially confirmed. Only 13% of the individualised goals of care were defined using these questionnaires, most of which focused on self-care activities and preparing beverages the patient enjoyed. Notably, only a single individualised goal of care related to the Environmental Factors category of the ICF was identified in our study. This absence could be attributed to the prioritisation of other goals during the acute neurorehabilitation phase, as environmental factors may become more relevant in the context of subacute or chronic management of functional impairments.

We propose that these individualised goals of care may be more challenging to document consistently in medical records compared to more standardised and repetitive objectives. This discrepancy could help explain the differences observed between the individualised goal of care analysis and the findings from the semi-structured interviews.

### 4.3. Insights from Semi-Structured Interviews

The semi-structured interviews highlighted the diverse uses of life history questionnaires in acute neurorehabilitation care. First, they are instrumental in building trust, not only with patients but also with their families and close social circles. Occupational therapists, responsible for gathering information from these questionnaires, noted that this process helps establish trust with family members. All eleven caregivers and therapists interviewed consistently described life history questionnaires as a valuable tool for “finding a gateway” to connect with patients.

Second, life history questionnaires promote multidisciplinary cohesion by providing a shared understanding of the patient’s identity. This “common vision” enhances collaboration and relationships among caregivers and therapists, strengthening interdisciplinary coordination and fostering a sense of unity across different therapies.

Lastly, life history questionnaires facilitate patient-centred care by fostering empathy and a sense of closeness with patients. Those interviewed reported observing improved patient responsiveness in this setting and expressed a sense of professional fulfilment, feeling that they were “doing their job right”.

However, the semi-structured interviews revealed discrepancies with the analysis of the individualised goals of care. These interviews highlighted an alternative use of the questionnaires in acute neurorehabilitation care. As noted in the results, the questionnaires play a key role in fostering a therapeutic alliance with patients and their caregivers, enhancing cohesion among the therapy team, and creating a more rewarding, patient-centred work environment for healthcare professionals.

#### 4.3.1. Therapeutic Alliance

The information from the life history questionnaires was effectively used to build relationships with patients by personalising interactions, such as addressing patients by their given names or nicknames, and discussing topics likely to “place the patient in a motivational frame and foster a trusting relationship”. These questionnaires were completed with the assistance of family members or caregivers within the first few days of hospitalisation in the acute neurorehabilitation ward. As highlighted in the introduction, the involvement of a patient’s close social circle is integral to the care process, particularly for non-communicative patients.

Acute neurorehabilitation patients endure a complex, multidimensional burden involving interconnected biopsychosocial and spiritual factors [42]. Addressing this burden requires a comprehensive approach that goes beyond medical care to encompass emotional and relational support. The interviews emphasised the critical role of understanding the family context, which, in many cases, proved vital for integrating relatives—and even pets—into the care process. Whenever possible, photographs of patients with their loved ones were placed at the bedside and utilised as a means of fostering communication. This approach sought to recreate a sense of familiarity and comfort for the patients.

A common feature across all acute brain injury aetiologies is the abrupt onset, leaving families unprepared and at significant risk of dysfunction. This can result in heightened levels of anxiety and depression. Early involvement of the family in the rehabilitation process is critical, as they will play an essential role in the patient’s subsequent reintegration into daily life.

Examples from the interviews illustrate the practical application of these questionnaires in defining individualised goals of care. One patient, who was known for preparing pancakes for his family, was encouraged to engage in this activity as part of his therapy, facilitating his social reintegration within the family unit. Another example involved a patient with a strong attachment to his dog. His life history questionnaire noted, “M. is very attached to the dog he has taken in”. This information guided the team in designing therapeutic sessions in the therapeutic garden, [26] where the patient could interact with his dog. This approach aimed to create a motivational framework for the patient by integrating meaningful and familiar activities into his rehabilitation plan.

#### 4.3.2. Multidisciplinary Cohesion

Cohesion and communication within the acute neurorehabilitation team are critical components of care, with significant implications for patient outcomes. The literature highlights that “cooperation in rehabilitation teams could be improved through better patient-oriented interprofessional communication” and that “informal communication is an important addition to formal communication” [43]. These findings align with the role of life history questionnaires, as reported in the interviews, which serve as a transversal and patient-centred tool to enhance team communication and collaboration.

For patients with limited communication abilities, there is a risk of speculative interpretations regarding their identity, the circumstances of their injury, or their “cardinal values” by different healthcare professionals. The life history questionnaires help mitigate these risks by providing a shared and consistent information base accessible to all team members. This unified reference reduces discrepancies in understanding the patient’s identity and context while helping avoid discussions of “sensitive topics” that may evoke traumatic memories for the patient. By preventing such conflicts, the questionnaires support a more cohesive therapeutic approach and promote smoother interactions during rehabilitation sessions.

#### 4.3.3. Patient-Centred Care and Empathy Promotion

As highlighted in the findings, patient-centred care—or patient-centred care by proxy, as is inevitably necessary in the unique context of unresponsive patients—enhances empathy among healthcare professionals by recognising patients as unique individuals with diverse backgrounds. This approach deepens understanding and strengthens the connection between healthcare providers and patients, fostering a compassionate and empathetic care environment [44,45]. This recognition enables caregivers to view the patient as an “individual”, preserving continuity in the patient’s identity despite the constrained communicative context imposed by lesions or altered states of consciousness. By encompassing details about hobbies, interests, life history, and personality, the questionnaires serve as a “snapshot” of who the patient was prior to hospitalisation. This can act as a reference point or an “ideal goal”, guiding the objectives of the acute neurorehabilitation management toward restoring functionality as closely as possible to the pre-injury state.

The “Personality” section of the questionnaires was frequently highlighted during the interviews. Acknowledging the patient as an individual with unique personality traits and a distinct life history, many caregivers adapted their therapeutic approach to align with the patient’s characteristics. For instance, a patient described as “strategic” and needing to understand the purpose behind tasks was treated accordingly. Therapists tailored their approach to focus on clear explanations, explicitly outlining the individualised goals of care and importance of therapeutic sessions. This personalised strategy helped engage the patient more effectively, emphasising the importance of individualisation in the rehabilitation process.

While existing research predominantly focuses on nursing, our interviews highlighted that this enhanced empathy was acknowledged by all healthcare professionals involved in acute neurorehabilitation, not only amongst the nursing team. This emphasises the impact of patient-centred care in nurturing empathy across diverse healthcare disciplines. While the direct retrospective influence of this approach on patient prognosis cannot be quantified, the consensus among the eleven caregivers and therapists was unanimous in appreciating a more “humane” care approach that facilitates empathetic and responsive patient care.

### 4.4. Limitations

Several limitations of the life history questionnaires have been identified. First, the subjective nature of the testimonies relies heavily on input from the patient’s close entourage, which may not always be available or entirely accurate. In the challenging post-traumatic context, family members and close friends may inadvertently provide a biased account, often portraying the patient in an overly virtuous light. Traits such as “fighting spirit” and “strength of will” may be exaggerated, while less socially desirable characteristics may be overlooked or omitted. Consequently, while the life history questionnaires aim to provide insight into the patient’s identity, they are not a precise tool, and pre-existing relational dynamics before the accident can influence the accuracy of the information recorded. Additionally, patients who are socially isolated or have a less present family environment may have incomplete life history questionnaires, limiting their usefulness in guiding care and therapy.

Our analysis also faced methodological constraints. Given the small number of patients treated in our highly specialised acute neurorehabilitation unit, this pilot study focused on qualitatively describing the implementation of life history questionnaires to complement individualised goals of care rather than statistically demonstrating their impact on rehabilitation effectiveness. Moreover, the integration of the questionnaires into care and therapy was not explicitly documented in medical records, preventing us from quantifying the extent to which they were used as tools to achieve individualised goals of care. This lack of documentation underscores the need for clearer and more consistent reporting practices to evaluate the impact of the questionnaires on patient care effectively.

## 5. Conclusions

Life history questionnaires developed for a specialised neurorehabilitation unit treating patients with clinical cognitive motor dissociation provide a comprehensive range of individualised information. They are essential for occupational therapists in defining ICF-derived individualised goals of care, potentially enhancing the neurorehabilitation process. For all other healthcare professionals involved, these questionnaires serve as a valuable resource, supporting the achievement of individualised care goals by qualitatively enriching the patient–provider relationship and shaping the therapeutic environment. However, they do not directly modify the individualised goals of care themselves. To validate the effectiveness of this tailored approach, a controlled study beyond this pilot investigation is warranted.

## Figures and Tables

**Table 1:** Patient characteristics and scores at admission and discharge from the acute neurorehabilitation ward. Patients are colour-coded the same way as in Figure 1.

Patient No.	Diagnosis	CRS-R		DRS		FAC	RLAS		Barthel		GOS
		Admission	Discharge	Admission	Discharge	Discharge	Admission	Discharge	Admission	Discharge	Discharge
1. ● ♂	MCS-, cCMD	12/23	21/23	19/29	7/29	3/5	2.5/10	6/10	−170/100	−55/100	3/5
2. ● ♀	MCS+, cCMD	15/23	21/23	18/29	7.5/29	1/5	5/10	7/10	−95/100	−10/100	4/5
3. ● ♂	MCS-, cCMD	12/23	15/23	21/29	18/29	1/5	5/10	5/10	−175/100	−170/100	3/5
4. ● ♀	MCS+, cCMD	14/23	20/23	22/29	11/29	1/5	3/10	5/10	−120/100	−95/100	3/5
5. ● ♂	MCS-, cCMD	11/23	23/23	21/29	9.5/29	0/5	3/10	6/10	−75/100	−45/100	3/5
6. ● ♂	eMCS, cCMD	20/23	23/23	14/29	3/29	4/5	6/10	9/10	−175/100	90/100	4/5
7. ● ♂	MCS-, cCMD	8/23	19/23	22/29	10/29	2/5	3/10	6/10	−175/100	−85/100	3/5
8. ● ♀	eMCS, cCMD	23/23	23/23	6/29	4/29	4/5	2/10	9/10	−25/100	85/100	4/5
9. ● ♂	MCS+, cCMD	14/23	eMCS *	12/29	7/29	1/5	5/10	7/10	−145/100	45/100	3/5
10. ● ♂	MCS-, cCMD	15/23	18/23	20/29	11.5/29	1/5	4/10	5/10	−125/100	−20/100	3/5

Barthel: Barthel Index, cCMD: clinical cognitive motor dissociation, CRS-R: Coma Recovery Scale-Revised, DRS: Disability Rating Scale, eMCS: emergence from Minimally Conscious State, FAC: Functional Ambulation Categories, GOS: Glasgow Outcome Scale, MCS: Minimally Conscious State, NA: not available, RLAS: Rancho Los Amigos Scale. * Exact CRS-R score not recorded.

## Data Availability

The dataset is only available upon request from the authors, due to the need for vetting to ensure anonymity.

## Supplementary Materials

The following supporting information can be downloaded at: https://www.mdpi.com/article/10.3390/brainsci15030267/s1, Table S1: Life history questionnaire, Table S2: Questions asked for semi-structured interviews, Table S3: Example of the implementation of life history questionnaires.

## Abbreviations

The following abbreviations are used in this manuscript:

cCMD	clinical cognitive motor dissociation
CMD	cognitive motor dissociation
CRS-S	Coma Recovery Scale-Revised
DOC	disorders of consciousness
DRS	Disability Rating Scale
EEG	Electroencephalogram
eMCS	Emergence from Minimally Conscious State
FAC	Functional Ambulatory Category
fMRI	Functional Magnetic Resonance Imaging
GOS	Glasgow Outcome Scale
ICF	International Classification of Functioning, Disability and Health
MCS	Minimally Conscious State
MCS-	Minimally Conscious State-Minus
MCS+	Minimally Conscious State-Plus
MBT-r	Motor Behaviour Tool-revised
RLAS	Rancho Los Amigos Scale
SMART	Specific, Measurable, Attainable, Realistic, Time-bound
UWS	Unresponsive Wakefulness Syndrome
