# Supplementary material for: The Role of Life History Questionnaires in Defining Individualised Goals of Care for Clinical Cognitive Motor Dissociation Patients: A Pilot Study
Source: Brain Sci. 2025 Mar 1;15(3):267. doi: 10.3390/brainsci15030267 (PMC11940304; doi:10.3390/brainsci15030267)
Supplement: Supplementary file 1 [file brainsci-15-00267-s001.zip › brainsci-3475837-supplementary.pdf]

brain sciences

MDPI

Supplementary Data

Table S1: Life history questionnaire (translated from the original French by Y.R.M. and I.A.M.).

**General Data**		
Name and surname:	Date of interview:<br>People involved and relationship with the patient:	
What is the patient's mother tongue?	Spoken	Written
Does he use other languages? (e.g., spoken, understood, read)		
What is his handedness?		
Does he have a visual impairment? (e.g., myopia, hypermetropia, color blindness, etc.)		
Does he have a hearing impairment?		
Does he use a hearing aid?		
**Entourage**		
1	Does the patient live alone? If not, with whom?	
2	Name, address, phone number of the partner/spouse<br>Other relevant information (e.g., profession)	
3	Names of the patient's significative relatives/children/close friends<br>Other relevant information (e.g., age, contact information, if deemed relevant)	
**Social and Professional History**		
4	Does the patient have a nickname?	
5	Where did he spend his childhood?	
6	What professional training has he undergone?	
7	What is his current/past job (if retired)?	
8	What past events/major social events can he remember? (e.g., marriage, birth of child, achievements)	
**Personality**		
9	What was the patient's personality like before the illness or accident? Please provide details if possible. If needed, include examples such as: optimistic, outgoing, quick-tempered, discouraged, curious, anxious, withdrawn, modest, sociable, irritable, impulsive, thoughtful, sensitive, rigid, conciliatory, or other traits.	
10	Does he usually react strongly (positively or negatively) to particular elements?	
**Spirituality**		
11	What are the patient's values, philosophy of life, convictions and beliefs?	
Questions 12 and 13 should be asked if considered relevant (depending on question 11).		
12	Does he have any practices related to his beliefs (e.g., reading, music, prayers, objects, body care)?	
13	Would it be important for the patient to be in contact with a representative of his faith community?	
**Personal Interests/Hobbies**		
14	Does the patient belong to any clubs or societies? Please provide details.	
15	Does he enjoy music?	
16	Does he play / Has he ever played a musical instrument? Please specify.	
17	Does he practice / Has he ever practiced a sport on a long-term basis? Please specify.	
18	Does he watch television?	
19	Does he have a computer/tablet? Specify usage.	
20	Does he have a cell phone? Specify usage.	
21	Does he listen to the radio? Specify the station.	

22	Does he enjoy reading? Please specify. (e.g., newspapers, magazines, books, authors)
23	Does he have any other hobbies or interests?
24	Are there any places that are significant/important to the patient? (e.g., nature, vacations)
25	What kind of food and tastes does he like/dislike?
26	Does he have a special diet?
27	What drinks does he like/dislike?
28	Does he/she have one or more pets? Please specify.
**Activities of daily living**	
29	What are the patient's personal hygiene habits (e.g., shower, bath, sink, frequency) and personal care routines (e.g., frequency of tooth brushing, type of toothbrush, use of makeup, frequency of shaving, type of razor)?
30	What kind of clothes/shoes does he wear?
31	Does he often feel cold?
33	Does he cook?
33	Does he usually do other household chores? Please specify.
34	Does he perform administrative tasks? Please specify.
35	Does he have his own rhythm or habits for eating (e.g., schedule, environment, position, quantity)?
36	What is his sleep/wake rhythm?<br>Does he take naps?
37	How does he get around? Does he drive a vehicle? If so, please specify. Does he use any mobility aids?
38	What type of home does he live in (e.g., house, apartment, floor, elevator, number of steps, architectural barriers)?

Table S2: Questions asked for semi-structured interviews

Were life history questionnaires employed in patient management?
If so, how were they useful?
If not, what were the reasons?
Did knowledge of the patient's life history, obtained through the questionnaires, influence the therapeutic relationship?
Did the life history questionnaires assist in formulating or achieving individualised goals of care?

Table S3: Example of the implementation of life history questionnaires

	Care	Therapeutic Sessions	Multidisciplinary Cohesion	Relationship Dynamics
**Information derived from history questionnaires**	"He listens to music on his device"<br>"Essential oils"<br>"Taking care of his dogs helped him get through his burn-out"	"He enjoys making pancakes for his family"<br>"He usually trims his beard with an electric razor"	"His wife allowed us to explain the circumstances of the accident so that the therapists are aware of it and avoid asking repetitively"	"He is Cartesian [transl. note: logical, analytical] and strategic. He needs to understand the meaning and logic of

				things in order to be motivated”
**Examples of implementation**	- Essential oils spraying on his shirt<br>- Display of his music<br>- Disposal of family and pets pictures in the room	Occupational therapy sessions assessing these activities	Discussed during the multidisciplinary meetings, recommendation of not addressing this subject during care and therapy	More in-depth explanations of the assessed activities
